# DistAMo: A Web-Based Tool to Characterize DNA-Motif Distribution on Bacterial Chromosomes

**DOI:** 10.3389/fmicb.2016.00283

**Published:** 2016-03-11

**Authors:** Patrick Sobetzko, Lukas Jelonek, Marc Strickert, Wenxia Han, Alexander Goesmann, Torsten Waldminghaus

**Affiliations:** ^1^Chromosome Biology Group, LOEWE Center for Synthetic Microbiology, SYNMIKRO, Philipps-University MarburgMarburg, Germany; ^2^Bioinformatics and Systems Biology, Faculty of Biology and Chemistry, Justus-Liebig-UniversityGiessen, Germany

**Keywords:** bioinformatics, computational biology, algorithm, chromosome maintenance, DNA replication, *Escherichia coli*, bacteria

## Abstract

Short DNA motifs are involved in a multitude of functions such as for example chromosome segregation, DNA replication or mismatch repair. Distribution of such motifs is often not random and the specific chromosomal pattern relates to the respective motif function. Computational approaches which quantitatively assess such chromosomal motif patterns are necessary. Here we present a new computer tool DistAMo (Distribution Analysis of DNA Motifs). The algorithm uses codon redundancy to calculate the relative abundance of short DNA motifs from single genes to entire chromosomes. Comparative genomics analyses of the GATC-motif distribution in γ-proteobacterial genomes using DistAMo revealed that (i) genes beside the replication origin are enriched in GATCs, (ii) genome-wide GATC distribution follows a distinct pattern, and (iii) genes involved in DNA replication and repair are enriched in GATCs. These features are specific for bacterial chromosomes encoding a Dam methyltransferase. The new software is available as a stand-alone or as an easy-to-use web-based server version at http://www.computational.bio.uni-giessen.de/distamo.

## Introduction

Chromosomes are much more than haphazard arrays of genes. Furthermore, they need to be physically and temporally coordinated during replication, segregation and systematically unfolded and refolded to fit in the cell. Such processes are collectively referred to as chromosome maintenance. Systems that are involved in chromosome maintenance often depend upon DNA motifs that are specifically bound by one or more proteins (Touzain et al., 2011; Messerschmidt and Waldminghaus, 2014). One example is the FtsK orienting polar sequences (KOPS) in bacteria which direct the DNA translocase toward the *dif* site opposite to the replication origin (Bigot et al., 2005). At this site FtsK interacts with the site-specific recombination system XerCD to resolve chromosome dimers. Another example is the nucleoid occlusion (Adams et al., 2014) in which a protein binds to specific sites on the chromosome and blocks cell division if the chromosome spans the division site. In this way the chromosome is protected from being guillotined.

Beside the individual DNA motifs and the binding protein there is a third aspect essential for the functionality of chromosome maintenance systems: the chromosomal distribution of the respective DNA motif. For the KOPS motif, a directional distribution was found on both replichors in the origin-to-*dif* site orientation (Bigot et al., 2005). For nucleoid occlusion, the motif is excluded from an extended region around the replication terminus, both in *E. coli* and *B. subtilis* (Wu et al., 2009; Tonthat et al., 2011). However, the functional relevance of this distribution is, to date, merely speculative. In other systems the chromosome-wide motif distribution was found to be directly linked to function (Touzain et al., 2011). In view of this development, the ever-expanding collection of sequenced genomes in recent years has been used for computational analysis of motif distributions. One example is the discovery of KOPS-like motifs in *Lactococcus lactis* by using three criteria derived from known KOPS (Nolivos et al., 2012). First, the over-representation in the genome, second, a leading strand bias and third, an especially high leading strand bias in the region around the *dif* site. The discovered motif in *L. lactis* was experimentally validated to be a functional KOPS.

Motif distribution analysis was also applied to find completely new chromosome maintenance systems. Mercier et al. hypothesized the presence of a dedicated protein organizing one chromosomal domain in *E. coli* and predicted that a respective DNA binding motive is over-represented specifically in this domain (exceptionality score) compared to the rest of the chromosome (contrast score). Plotting the two values against each other for all possible 11-mers revealed a novel motif (matS) which was found to interact with a protein (MatP). This combination contributes to organization of the Ter macrodomain (Mercier et al., 2008).

Although the described computational methods led to interesting new biological insights the focus was not on a detailed and systematic analysis of the chromosome-wide distribution of the respective DNA-motifs. It is actually not trivial to determine if a DNA motif occurs at a specific site only by chance or if the motif is over- or underrepresented at a locus (Sadovsky, 2006). The critical point is that over- or under-representation is by definition relative to the so called null-model. The most common approach is to calculate the occurrence of sub-motifs and from that derive the likelihood of them to form the motif itself. The logic is that if there are, for example, many GA and TC dinucleotides in a sequence the chance of a GATC would increase. The expected incidence of GATC is relative to the number of GAs and TCs. Thus, a single occurrence of GATC in a region with many GAs and TCs would not be considered an over-representation. Conversely, in a region in which the only GA and TC dinucleotides formed a GATC motif, this incidence would be considered an over-representation. The problem with this approach is that it does not take into account the rules and constraints that might apply to biological sequences. As a way to include the biological characteristics of the sequences into the motif distribution analysis we use the codon redundancy as a basis for our calculations (see Results section for a detailed description).

Implementation and application of the respective algorithm revealed new insights on the functional important sequence motif GATC. This sequence is special in *E. coli* and related γ-proteobacteria because it is methylated at the adenine in both strands of this palindromic sequence by the Dam methyltransferase (Geier and Modrich, 1979). This methylation is important for different cellular processes (Løbner-Olesen et al., 2005). Firstly, the methylation contributes to the efficient repair of mismatch mutations. This is because freshly replicated DNA will be methylated on only one strand (the old strand) and unmethylated on the other (the new strand). To repair a mismatch one of the unpaired nucleotides is excised and replaced by a complementary nucleotide. The role of methylation is to direct the repair to the new strand via the protein MutH that binds specifically to hemi-methylated GATCs. A second protein that binds hemi-methylated GATCs specifically is SeqA (Waldminghaus and Skarstad, 2009). SeqA was found as factor that sequesters the origin of replication, *oriC*, after initiation of DNA replication (Lu et al., 1994). In addition, SeqA binds to a stretch of DNA behind the replication fork and was suggested to contribute to chromosome segregation (Waldminghaus et al., 2012; Joshi et al., 2013). In addition to its role in mismatch repair, DNA replication and chromosome segregation, the GATCs have also been shown to be involved in gene regulation (Casadesús and Low, 2006). The best studied example is in phase variation in pathogenic *E. coli* strains (Blyn et al., 1990). The multiple roles of GATCs in bacterial cells make it an attractive target for detailed analysis of its distribution on bacterial chromosomes. We therefore used our new computer tool DistAMo to uncover significant distribution patterns of the GATC motif within single genes, multiple genes grouped according to function, and whole genomes, demonstrating the versatility of DistAMo.

## Materials and methods

### The DistAMo algorithm

**Figure 1:**
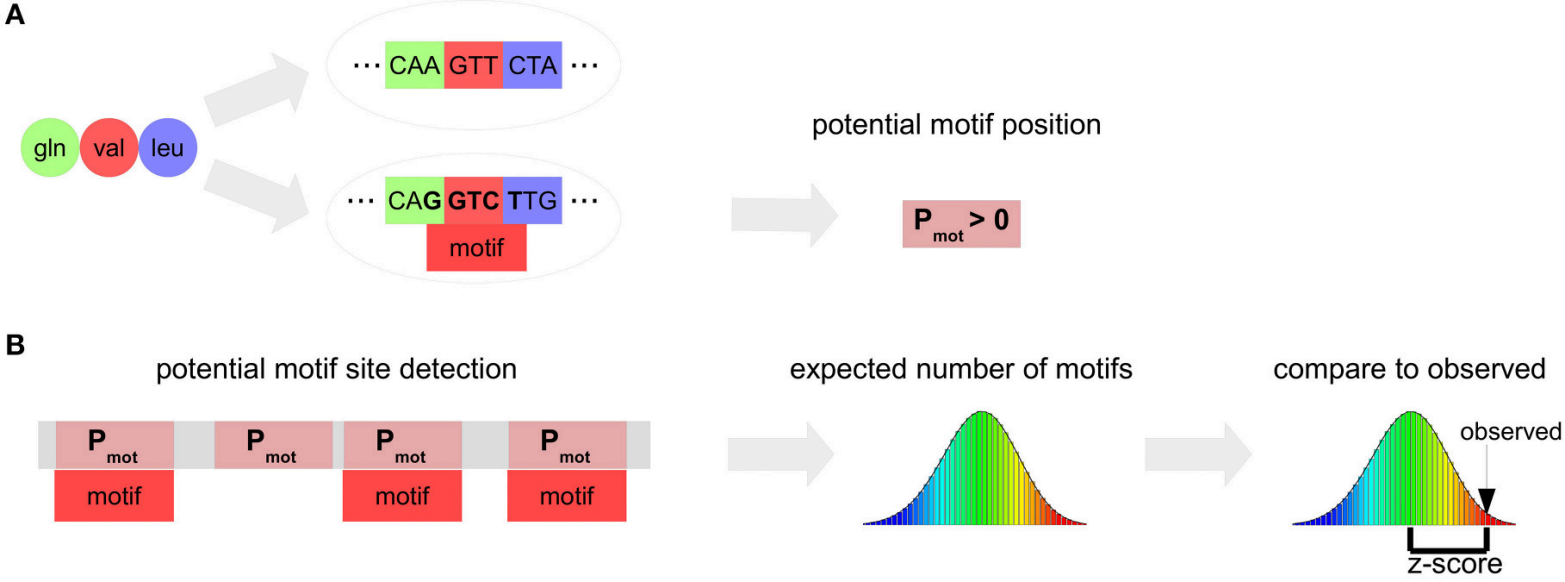
**Scheme of the motif analysis algorithm DistAMo. (A)** The amino acid sequences encoded by a motif-containing DNA sequence (pot motifs) are determined. A probability of each potential motif to be encoded by a motif containing sequence is calculated. **(B)** The positions of potential motifs are detected using a suffix tree search in the proteome and assigned to the corresponding genes, gene groups or chromosomal region depending on the type of analysis. The random distribution of the number of motifs follows a Poisson binomial distribution. The z-score (significance value) is determined from the actual number of motifs, the mean (the expected number of motifs) and the standard deviation of the Poisson binomial distribution.

The DistAMo algorithm first determines amino acid combinations (*pep*) that form a potential motif. A potential motif is a position in a coding region where the encoded amino acids allows for a certain motif to occur (Figure 1). The length of each *pep* is defined by the number of codons that the motif of length *l* can span and is determined by the following rule:

$$\begin{array}{l} \;\left\lfloor \frac{l}{3}\right\rfloor ,\;\left\lfloor \frac{l}{3}\right\rfloor +1\;l\text{ mod}3=0\\ \;\left\lfloor \frac{l}{3}\right\rfloor +1\;l\text{ mod}3=1\\ \left\lfloor \frac{l}{3}\right\rfloor +1,\left\lfloor \frac{l}{3}\right\rfloor +2\;l\text{ mod}3=2 \end{array}$$

As example, the dipeptide Arg-Ser forms a potential motif for the DNA motif GATC, as Arg Ser can be encoded by A**GA TC**C. The probability *P*_*mot*_ of the motif to occur in the coding sequence for an amino acid sequence *pep* is defined by the sum of frequencies of coding sequences *cod*_*mot*_ containing a motif and coding for the amino acid combination over the sum of frequencies of sequences *cod* coding for the amino acid combination. Coding sequence frequencies are derived from all coding sequences.

$$\begin{array}{l} P_{mot}\left(pep\right)=\frac{\sum f\left(cod_{mot}|pep\right)}{\sum f\left(cod|pep\right)} \end{array}$$

Taking the example of GATC motifs, the genome-wide frequency of AGA TCC would be one of the coding sequence frequencies f(*cod*_*mot*_*|pep*) summed up in the nominator. The genome wide frequency of AGG TCT also encoding Arg Ser would be one of the coding sequence frequencies f(*cod|pep*) summed up in the denominator. After this step, we have determined all amino acid sequences that may form a potential motif. Using a suffix tree, the proteome is efficiently scanned for amino acid sequences that form a potential motif. With the potential motif and the probability for a motif occurrence at the potential motif we can directly obtain the expected number of motifs *m* and the standard deviation s for a given protein to determine a significance value (z-score; *z*) for the deviation of the number of observed motifs *m* from the expected number of motifs. Assuming independence of potential motifs in a coding sequence, the occurrence of motifs follows a binomial process with varying probabilities. The number of expected motifs therefore follows a Poisson binomial distribution with

$$\begin{array}{lll} \bar{m} & = & \sum P_{mot}\left(pep\right)\\ s & = & \sqrt{\sum P_{mot}\left(pep\right)\left(1-P_{mot}\left(pep\right)\right)}\\ z\left(m,\bar{m},s\right) & = & \frac{m-\bar{m}}{s} \end{array}$$

The approach can be extended to a set of proteins by merging the potential motifs (lists of probabilities) and computing the z-score as described above. With this flexibility and sophistication it is possible to approach specific biological questions including the investigation of motif distributions in a spatial and functional context.

## Results

### A new algorithm to analyse DNA motif distributions on bacterial chromosomes

For a biologically meaningful evaluation of motif abundance it is important to apply a null-model to distinguish between conspicuous accumulations of motifs and those arising by chance. Previous null-models were based on Markov-chains of various orders. Markov-chains take base neighborhood dependencies of nucleotide occurrence throughout the genome into account. However, chromosomal DNA is highly diverse. Using the complete chromosomal DNA sequence merges coding and non-coding DNA characteristics in the process of determining k-mer frequencies. Especially the non-coding DNA is highly diverse due to its manifold roles in transcriptional regulation (e.g., promoter sequences, terminators, and other regulatory sites). Hence, a one-fits-all approach is not recommended to perform reliable motif analysis. We therefore concentrate on the coding sequence for two reasons. First of all, it is the dominating sequence type in bacteria, covering approximately 90% of the total chromosomal DNA sequence (Land et al., 2015). Secondly, there is a single principle, the coding for proteins, dominating the evolution of this type of sequence. This allows the coding information of the sequence to be used as null model for the calculation of motif abundance. Such an approach would thus focus on the biological constraints of a sequence rather than on its pure statistics. We therefore propose the DistAMo (Distribution Analysis of DNA Motifs) algorithm that estimates the motif distribution by the coding flexibility of the protein coding DNA (Figure S1). This allows for a precise assessment of motif enrichment taking into account the protein coding information. An important term in this context is the potential motif. By our definition a potential motif is a position in a coding region where the encoded amino acids allows for a certain motif to occur (Figure 1). For example the motif GGTCT is possible when the peptide of Gln, Val, and Leu is encoded. On the other hand, these amino acids could also be encoded by other codons not leading to a GGTCT. The ratio between this potential motifs and the actual motif occurrence is the general value our novel algorithm is based on (details are provided in the Material and Methods section).

### Evaluation of motif-distribution interdependencies

**Figure 2:**
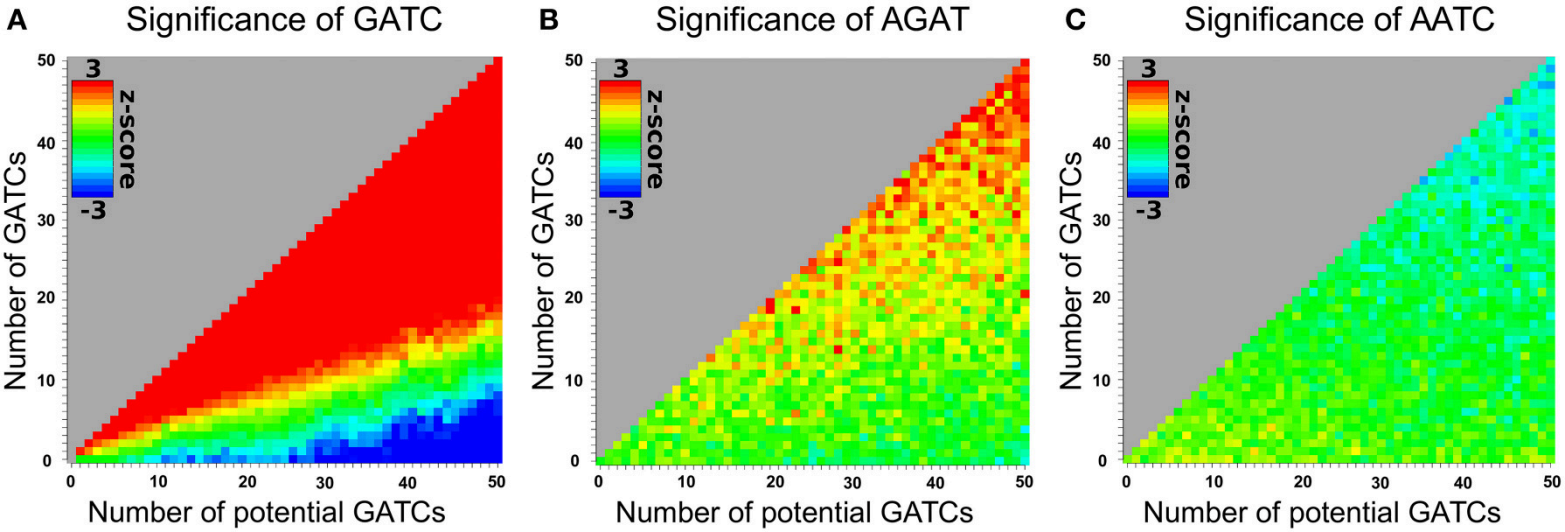
**Impact of motif and potential motif frequencies on the z-scores of other motifs**. The abscissa and ordinate show the number of potential GATC sites and real GATC sites respectively in an otherwise randomized 3000 bp coding sequence. The z-score for the tetramer is indicated in rainbow colors with red for a z-score ≥ 2 and blue for a z-score ≤ −2. **(A)** GATC z-scores. **(B)** AGAT z-scores for different enrichments of GATC (see axis). AGAT overlaps with GATC. An increase of GATC therefore increases the frequency of AGAT **(C)** AATC z-scores for different enrichments of GATC (see axis). AATC competes with GATC sites due to the sharing of potential motif sites. Therefore, an increase of GATC decreases the abundance of AATC.

**Figure 3:**
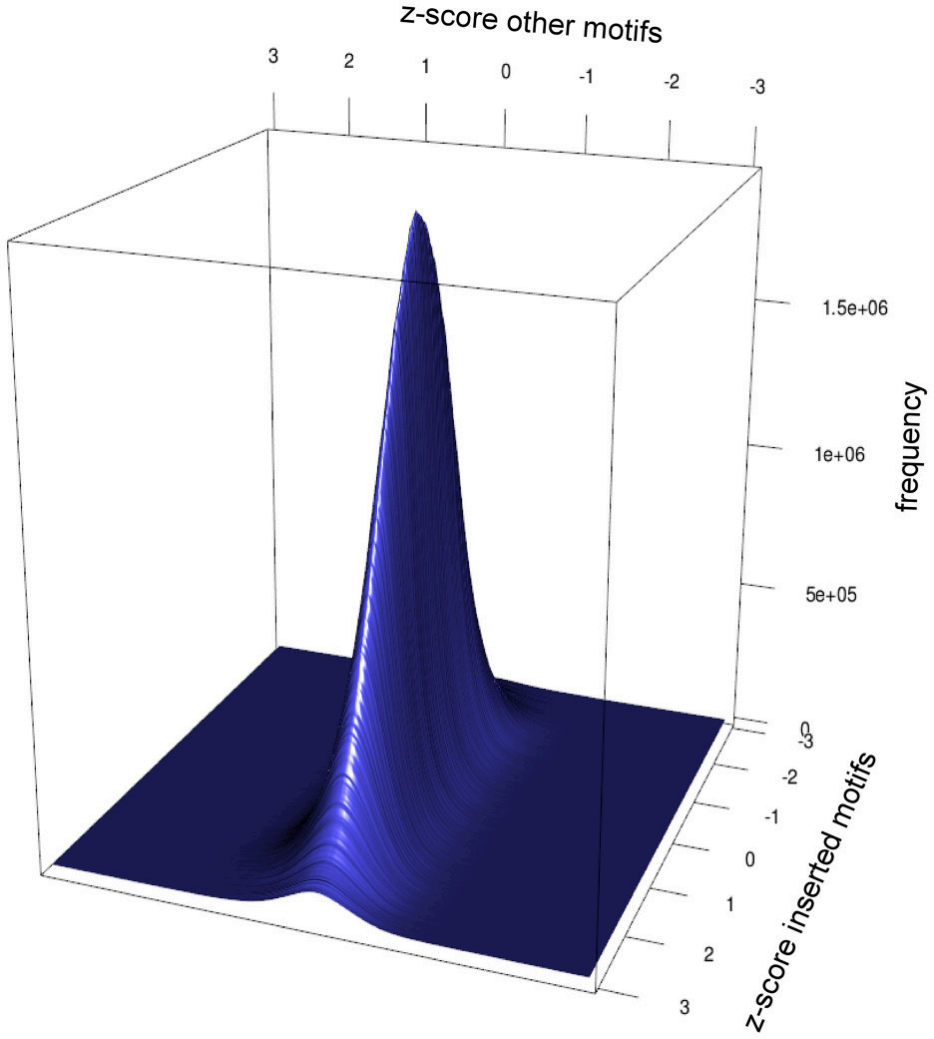
**Impact of motif enrichment on the z-scores of other motifs**. Depicted is the frequency distribution of z-score pairs, consisting of z-score of the enriched motif and the z-score of another motif in a random protein sequence. The distribution shows no dependence of an enrichment of tetramers on the z-score of other tetramers. In genes with random sequence approximately 1 out of 1000 genes show a significant enrichment for a tetramer if another tetramer was enriched significantly. This is equal to the frequency of a significant enrichment of a tetramer in a random sequence (P (A|B) = P (A)). Hence, within the limits of motif distributions present in bacteria no interference of tetramer z-scores are to be expected using DistAMo.

Motifs can't necessarily be looked at independently. Motifs can be prefixes or suffixes of other motifs, overlap, or share the same potential motif sites. To estimate the degree of interdependence and the impact on DistAMo results we performed a comprehensive study of tetramer interdependencies. The goal was to analyse the interdependence that stems from motif similarities and amino acid coding properties but not biological co-occurrence of motifs, as the detection of such biological signals is the aim of the tool. Hence, the analysis of motif interdependence cannot be done using native sequences as they may contain various numbers of motifs that are coupled for biological reasons. We therefore generated random coding sequences with a wide range of potential motifs and real tetramer motifs. For these sequences the z-scores for one tetramer with a defined number of occurrences was determined relative to the occurrence of other tetramers. Figure 2A depicts the z-scores of coding sequences with different numbers of GATCs. Figures 2B,C show the effect of potential and real GATCs on the occurrence of AGAT (part of GATC) and AATC (excluding GATC). It is apparent that at a certain number of motifs other motif scores are affected, which might trigger wrong conclusion. Over-representation of a motif of interest could for example only be a result of significant under-representation of another motif. To see how relevant this finding is for calculations based on natural sequences we analyzed z-scores of genes with motif distributions as found in the *E. coli* genome. We sampled random genes with motif and potential motif numbers of every gene in *E. coli* for every tetramer and applied DistAMo to all sequences searching for all tetramers. We therefore generated a set of genomes with a single tetramer represented like in native *E. coli* genes. Each of these artificial genomes was then analyzed regarding the abundance of each of the 256 possible tetramers. Figure 3 depicts the dependency of the z-score of the motif inserted in the random sequence and any other motif. The probability to show a significant z-score given a significant enrichment/depletion (z-score ≥ 2) is equal to the probability to show a significant z-score (P (mot_2_ sign |mot_1_ enriched/depleted) = P (mot_2_ sign) ≈ 0.001), indicating independence of motif z-scores. It becomes clear from our analysis that the z-scores of the other motifs are not affected by the z-score of a significantly enriched or depleted motif. Hence, within the range of biological motif distributions there is no danger of strong interdependencies of motif z-scores. DistAMo z-scores can therefore be regarded as independent. It is important to note that this should also hold true for DNA motifs longer than the tetramers used for the analysis here. This is because results are not related to sequence length but rely on the degree of motif similarity between the DNA motif of interest and the potentially interfering motif. Notably, the tetramer analysis includes all degrees of similarity from very similar to not similar at all.

### Implementation of DistAMo (distribution analysis of DNA motifs)

**Figure 4:**
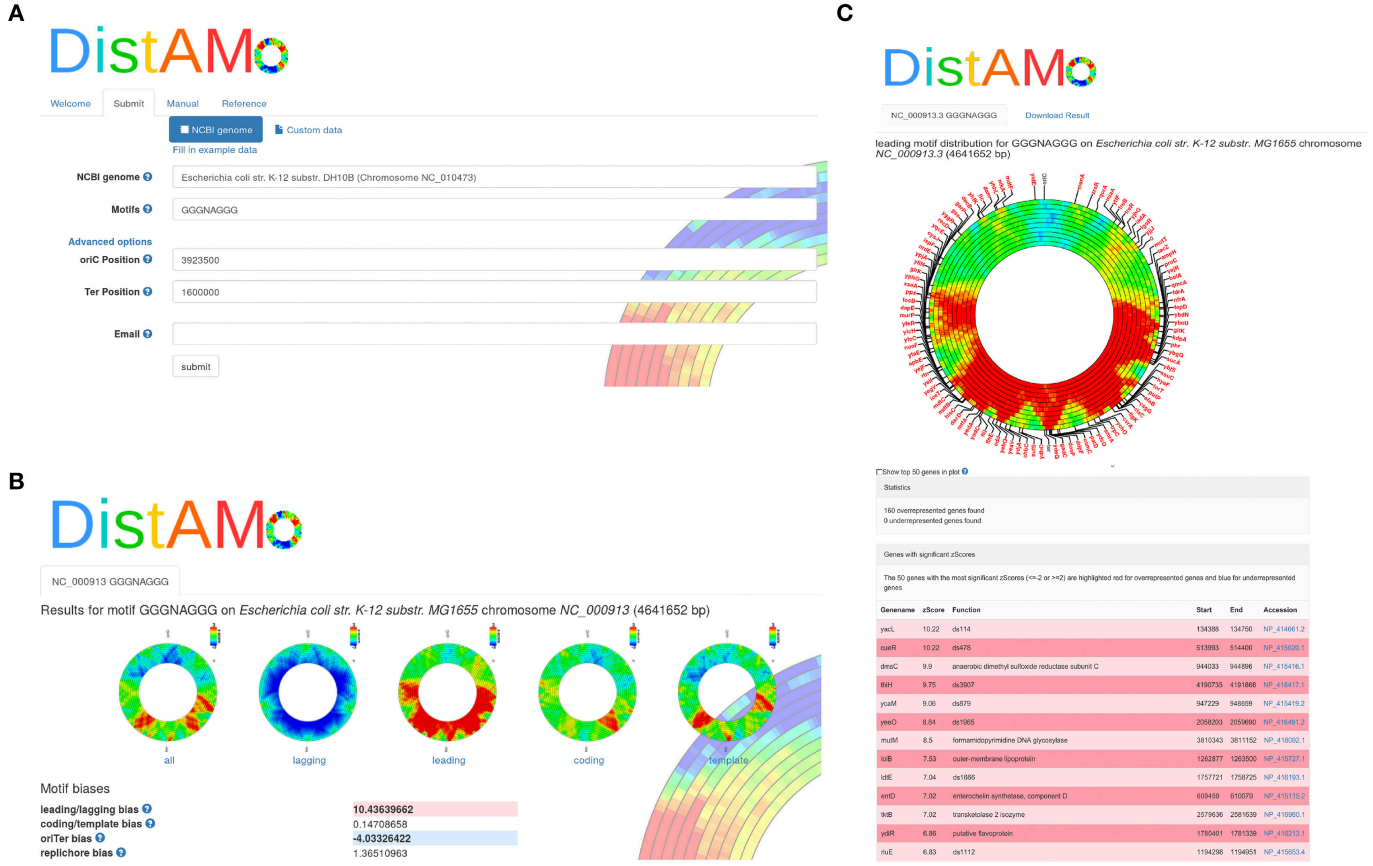
**DistAMo online version**. The tool is available online with a user-friendly interface to allow access also to non-experts. **(A)** Input mask where the user can choose from thousands of complete genomes and search for the motif of interest. Help symbols guide through the input. **(B)** After computation the user is informed via email and guided to the results. It holds global information including strand bias and ori-ter bias significance scores of the motif. **(C)** A click on each figure opens a page with detailed information about the genes with significant over- and under-representation. Gene positions can be displayed on the chromosome plot.

**Figure 5:**
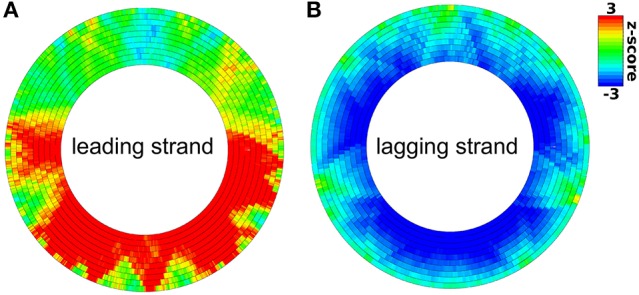
**Distribution of KOPS sites on the leading strand (A) and lagging strand (B) of the *E. coli* chromosome**. A significant over- or under-representation is color-coded by a red or blue color respectively. Rings from outside to the inside differ in the size of the sliding window from 50 to 500 kb in 50 kb steps.

The DistAMo algorithm was implemented and is available as stand-alone program or easy-to-use online tool (http://www.computational.bio.uni-giessen.de/distamo). Annotated genome information in fasta and a gff format together with a motif of interest serves as input to the program (Figure 4A). The online tool allows easy selection of chromosome sequences from an implemented list of about 7000 available replicons from bacteria, archaea, and viruses. A detailed manual and example data are provided to introduce researchers to DistAMo functionalities. The output of DistAMo is a list of z-scores describing the over- or under-representation for each coding sequence. In addition, the data is plotted color-coded in a set of circles with different moving window sizes to allow easy and fast visual inspection of the genomic motif distribution (Figures 4B,C, 5).

Many DNA motifs that are known to be functionally important show specific biases for regions of the chromosome. To get significance values for such biases the DistAMo program calculates z-scores for five relevant parameters: (i) the leading/lagging strand bias describes over-representation of the motif on either the leading or the lagging strand (ii) the coding/template strand bias measures the over-representation of the motif on the strand that corresponds to the coding mRNA vs. the strand that serves as mRNA template (iii) the origin/terminus bias compares motif enrichment in the chromosome half of the replication origin vs. the half containing the replication terminus, (iv) the replichore bias compares the motif enrichment on the left and right replichores, and (v) the subset bias compares the motif enrichment in a given subset of genes vs. the entire genome (available only in the stand-alone version).

The leading/lagging strand bias is determined using a Monte-Carlo simulation as follows. In the first step, the motif z-scores of all genes are computed separately for the leading and the lagging strand using the motifs and potential motifs of the respective strand. Then DistAMo determines the average difference of the z-score between leading and lagging strand of all genes. In the final step this difference is computed with randomized orientations (randomized leading/lagging strand) of genes and the mean and standard deviation of the difference of 10,000 replicates is determined to obtain a z-score. Calculation of the other biases works equivalently. They are not calculated for viral and archaeal genomes because their replication mechanism are more complex not allowing easy differentiation between for example leading and lagging strand. For bacterial genomes, DistAMo also generates genomic plots for motif distributions on the leading strand, the lagging strand, the template strand and the coding strand (Figure 4B). To test our algorithm with a known motif distribution we used KOPS sites (GGGNAGGG), known to be biased in leading/lagging strand distribution in *Escherichia coli* (Bigot et al., 2005). Genomic plots show a clear over-representation of KOPS on the leading and an under-representation on the lagging strand in *E. coli* as expected with a highly significant bias z-score of 10.3 (Figure 5). Interestingly, we also detected a biased distribution of KOPS along the ori-ter axis with a significant z-score of -3.8. Similar to *Lactococcus lactis*, these result indicates that KOPS are significantly enriched in the ter half of the *E. coli* chromosome (Nolivos et al., 2012).

### Genome-wide GATC distribution follows a distinct pattern

**Figure 6:**
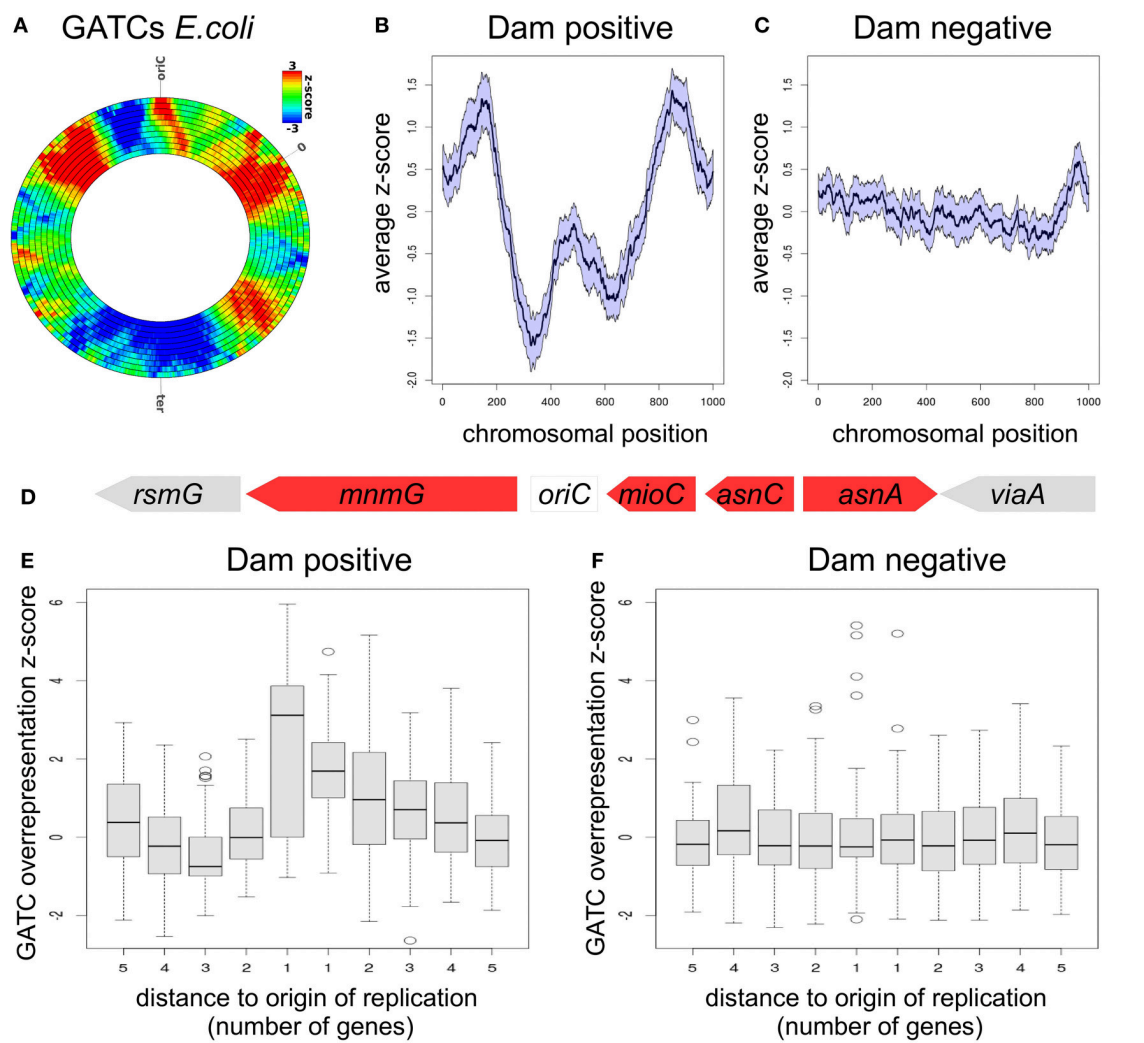
**GATC-distribution analysis with DistAMo**. **(A)** Rings depict the standard DistAMo output with different sliding window sizes (compare Figure 5) for the distribution of GATC sites in *E. coli*. Raw data are provided in Table S2. **(B)** Average z-scores of GATC densities on chromosomes of Dam positive γ-proteobacteria. Error bar (SEM) are indicated by the area around the curve. The origin of replication is situated in the 0 and 1000 position (circular chromosome). The z-score data of different-sized chromosomes were scaled to 1000. **(C)** Analysis as in **(B)** with Dam negative γ-proteobacteria. **(D)** *oriC*-proximal genes in *E. coli*. Overrepresentation of GATC is indicated by a red color. The set of γ-proteobacteria was split in Dam-positive and Dam-negative species and respective z-scores of *oriC*-proximal genes plotted **(E,F)**. Used species are listed in Table S1. GATC densities for *oriC*-proximal regions of all analyzed bacteria is shown in Figure S3.

After proving the effectiveness of DistAMo and establishing reproducibility of previous findings, we turned to the investigation of the genome-wide GATC distribution in *Escherichia coli*. The GATC over-representation peaked symmetrically at approximately one third of the distance to the terminus region on both replichores (Figure 6A). Raw data are provided in Table S2.

In order to identify a potentially conserved pattern of GATC distribution, the same analysis was performed with chromosome sequences of 152 γ-proteobacteria available on the NCBI server making sure that only a single genome per species is selected and only species with an *oriC* position listed in the D*oriC* database (Gao et al., 2013). The full list of species and a phylogenetic tree of the used γ-proteobacteria is provided in the Supplementary Material (Figure S2, Table S1). The functional importance of the GATC motif in *E. coli* is directly linked to the Dam methyltransferase which methylates the respective adenine specifically. Dam is evolutionary conserved in a subset of the γ-proteobacteria. In order to have a control distribution for GATC we split the 152 species into Dam positive and Dam negative species, representing functional and non-functional GATC motifs, respectively. However, Dam orthologs are difficult to identify by *in-silico* approaches due to similarities with non-Dam DNA methyltransferases. SeqA is co-conserved with Dam (Marinus and Lobner-Olesen, 2014) and can be detected reliably by BLAST searches. We therefore split the two sets using the *seqA* gene as an indicator of the presence of Dam. The list of γ-proteobacteria comprised 79 Dam positive and 73 Dam negative species (Figure S2, Table S1). We computed the average pattern of GATC distribution using scaled chromosome data to deal with the different sizes of chromosomes in the averaging process. This scaling approach has been successfully applied to γ-proteobacteria genomes in previous studies (Sobetzko et al., 2012, 2013). Our analysis revealed the conservation of the symmetric high-density regions found for *E. coli* in Dam positive γ-proteobacterial chromosomes (Figure 6B). In contrast, an even distribution of GATCs without distinct cluster patterns was observed for Dam negative chromosomes (Figure 6C). These findings suggest that the chromosome-wide distribution of GATCs is directly linked to the presence of *dam* on the respective genome.

### Genes beside *oriC* are enriched in GATC sequences

GATC sequences can be found with a high frequency in the origin of replication of *Escherichia coli*, reflecting the importance of GATC methylation for the proper function of *oriC*. Zooming into our GATC density analysis of coding regions near the origin shows an over-representation of GATCs directly adjacent to the *oriC* (Figure 6D). To investigate if this over-representation is conserved we analyzed the genes neighboring the origin in both Dam positive and negative sets of γ-proteobacteria described above (Figures 6E,F). Both genes directly neighboring the replication origin show a significant over-representation of GATCs with mean z-score values of 3.3 and 1.8, respectively. Thus, the GATC over-representation found at the replication origin includes the coding regions of adjacent genes (Figure 6E). Notably, this finding applies only to bacteria encoding a Dam homolog while no GATC enrichment was found for origin-neighboring genes in genomes of Dam-negative bacteria (Figure 6F).

### Genes involved in DNA replication and repair are enriched in GATCs

**Figure 7:**
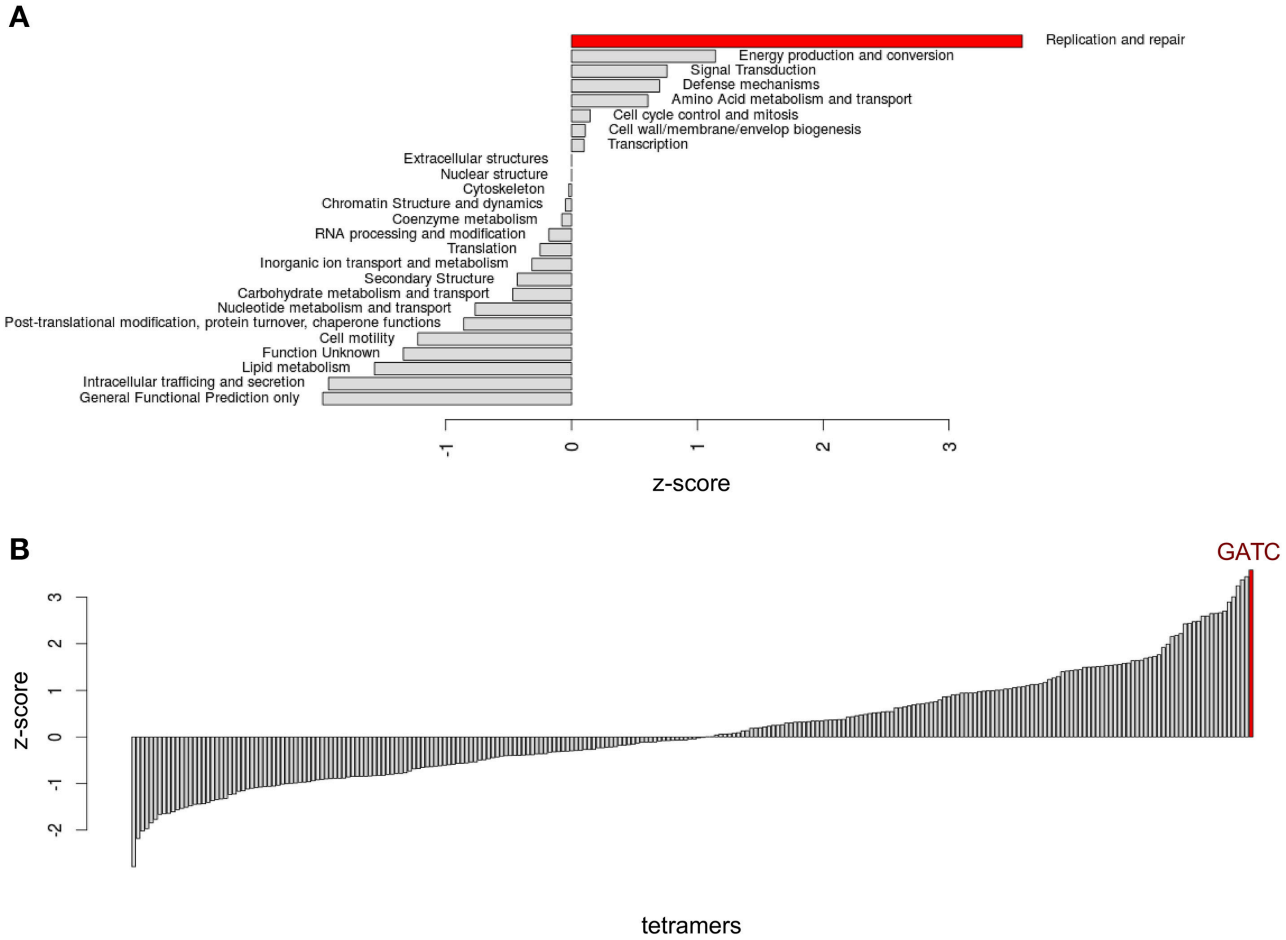
**Distribution of GATC in genes with specific functions**. **(A)** Analysis of GATC over-representation in *E. coli* for COG groups. **(B)** Calculation of over-representation for all tetramers in the most GATC over-represented COG group (Replication and repair).

In *Escherichia coli*, several other genes, in addition to the *oriC*-flanking genes, show a strong enrichment of GATC sites in their coding sequences. We asked whether these genes are functionally related and investigated COG (conserved orthologous gene) groups comprising *E. coli* genes belonging to the same functional class. Application of DistAMo provided z-scores for the over-representation of the GATC motif in these groups (Figure 7A). Interestingly, the group of replication and repair genes yielded the only significant (3.9) score of all groups (Figure 7A) with a large gap to the second highest score (1.2). To cross-check the gene group of replication and repair for the specificity of GATC over-representation we analyzed the over-representation z-score of all tetramers in this group (Figure 7B). Notably, GATC was most over-represented in this COG group among all 256 tetramers (Figure 7B) further supporting the significance of our finding.

## Discussion

Several algorithms determining the over-representation of motifs have been developed (Merkl et al., 1992; Karlin et al., 1994; Mrázek et al., 2002; Kural et al., 2009; Davenport and Tümmler, 2010; Schbath, 2011; Ding et al., 2012). Most of them are based on Markov-chains that lack the capability to differentiate between motif selective coding and other selective pressures on coding sequences (Kural et al., 2009). For the estimation of a motif over- or under-representation it is important to take into account the protein coding information level of a DNA sequence. A published algorithm does actually calculate over-representation of a motif based on the redundancy of the genetic code as we do in the work presented here (Ding et al., 2012). However, this algorithm is limited to the determination of a global over-representation in a given (large) sequence (Ding et al., 2012). Output of the respective program is a single value indicating the global over- or under-representation of a motif. Since the statistics for this determination is derived from the input sequence itself, small sequences like genes cannot be analyzed individually by this tool. In addition, local variations of the motif distribution would be partially occluded by the overall genomic over-representation of the motif in such approaches. Such local variations of motif distributions however, have been shown to be critical to understand cellular mechanisms related to chromosome maintenance (Mercier et al., 2008; Wu et al., 2009; Touzain et al., 2011; Nolivos et al., 2012).

The DistAMo algorithm introduced here provides rich information about the motif distribution among single genes, groups of genes and the whole genome with minimal input from the thousands of available genomes in a standard flat file format. With our user-friendly web-based version, the tool is openly accessible to regular biologists without a bioinformatics background. Although we focus on bacterial chromosomes our new algorithm is widely applicable also to other genomes. DistAMo will allow for example the analysis of DNA motifs in archaea and viruses where the genomes mostly consist of coding sequences as in bacteria. All available genome sequences (>500 kbp) of these three phylogenetic groups are included in the online version of DistAMo. For eukaryotic organisms, DistAMo could be used for the analysis of DNA motif densities in individual or groups of genes.

Application of DistAMo to the DNA motif GATC revealed three remarkable insights on its distribution on the *E. coli* chromosome and in the phylogenetic group of γ-proteobacteria. The first finding was that the GATC density follows a distinct pattern on the chromosome-wide scale (Figures 6A–C). Occurrence of this pattern was limited to chromosomes of bacteria that encode a SeqA homolog indicating that it is related to the Dam methylation system. What function might the specific genomic distribution of GATCs on the Dam-positive bacteria serve? The distribution might be associated with a protein of the so called *dam* clade (Brézellec et al., 2006; Marinus and Lobner-Olesen, 2014). This is a group of proteins which is evolutionary conserved with Dam. We have described MutH and SeqA above with their specificity for hemi-methylated GATCs which make them dependent on a functional Dam homolog. Other members of the Dam clade, such as the MatP protein or MukBEF do not have an obvious direct connection to Dam methylation (Niki et al., 1991; Mercier et al., 2008). The observed GATC pattern shows some symmetry with respect to the origin to terminus axis (Figure 6B). Such symmetry might point to some function related to DNA replication which proceeds bidirectional from ori to ter. DNA mismatch repair mediated by MutH might be such a process because it continuously proceeds on newly replicated DNA behind the replication fork. However, mismatch repair seems to be functional in regions with an under-representation of GATCs as long as a certain distance between neighboring GATCs is not exceeded, and does not increase in efficiency with increasing GATC density (Bruni et al., 1988). Even if this was the case the question remains why the mismatch repair should work with different efficiencies in different regions of the chromosome. Another Dam-clade protein associated with DNA replication is SeqA (Waldminghaus and Skarstad, 2009). It was discovered as factor that sequesters the replication origin *oriC* from inappropriately early rounds of re-initiation (Lu et al., 1994). Sequestration is mediated by binding of SeqA to the hemi-methylated GATCs that occur at *oriC* in high density. Such sequential binding of SeqA hinders the chromosome replication initiator protein DnaA from directly rebinding to *oriC* after each successful initiation of DNA replication. In addition to its role in origin sequestration, SeqA was found to bind dynamically to a stretch of newly replicated DNA following the replication fork (Waldminghaus et al., 2012). The mechanism by which SeqA leaves *oriC* after the sequestration period is unknown. One possibility is that titration contributes, where SeqA molecules are attracted by GATCs to the replication fork and in this way directed away from the replication origin. In that case, the strength of titration should consequently be linked to the density of GATCs in the region of the chromosome that is replicated at a respective time point. According to our data, titration strength would gradually increase from the time point of initiation until about one third of the chromosome is replicated (Figure 6A). The subsequent decrease in the GATC density could then gradually reduce the number of SeqA molecules at the replication fork to make them available for the next round of origin sequestration. The main problem with the outlined model is that origin sequestration periods vary greatly in *E. coli* due to the ability to grow with overlapping cycles of DNA replication. In contrast, the time point of replication forks reaching the genomic maximum of GATC density after initiation will be relatively constant since replication speed is relatively constant. Manifestation of SeqA titration strength in the GATC distribution on the chromosome might thus only be possible if it reflects the dominant growth pattern of respective bacteria.

The second interesting finding regarding GATCs in *E. coli* and related bacteria was that genes neighboring the replication origin show significant over representation (Figures 6D–F). This finding for *E. coli* appears to be conserved within Dam-clade bacteria while no GATC enrichment was found in genes beside the replication origins of other bacteria within the γ-proteobacteria. Why would there be a selection pressure for GATC enrichment near the replication origin? As for the chromosome-wide GATC pattern discussed above there might be a connection to the SeqA protein. The methylation of GATCs at *oriC* of *E. coli* was shown to persist following replication much longer than elsewhere on the chromosome (Campbell and Kleckner, 1990). This was attributed to the high density of GATCs at *oriC* itself and a respective multimerization of SeqA that is more stable compared to individual SeqA dimers. In fact, *oriC* had the highest signal of all SeqA binding sites in ChIP-Chip experiments (Sánchez-Romero et al., 2010; Waldminghaus and Skarstad, 2010). It might thus be reasonable to conclude that the high density of GATCs in origin-neighboring genes increase the binding strength of SeqA to the origin itself. Direct support for this assumption comes from an experiment where synthetic clusters of GATCs where introduced to different sites on the *E. coli* chromosome leading to increased SeqA binding at nearby sites (Waldminghaus et al., 2012).

The third finding on GATC enrichment from this study is most puzzling. It appears that genes involved in DNA replication and repair show significantly higher GATC densities compared to all other functional categories and that no other tetramer shows higher over-representation in DNA replication and repair genes than GATC (Figure 7). We have outlined above that the most of what we know about GATC and its function within the cell is related to DNA replication and repair. But why should these genes have a high GATC density? One might intuitively suspect some sort of gene regulation. Others have indeed considered the existence of a GATC regulon that might consist of genes with high numbers of GATCs in their coding region (Riva et al., 2004a,b; Sánchez-Romero et al., 2010). However, global transcription analysis of SeqA or Dam mutants gave no clear indication for such a regulon (Oshima et al., 2002; Lobner-Olesen et al., 2003).

In conclusion we have found three new insights on GATC distribution in γ-proteobacteria which are obviously linked to Dam and co-evolved genes. Our data strongly suggest that there is a significant selection pressure associated with the GATC densities, suggesting their importance for survival. The inability to find easy explanations might indicate that some completely new mechanism remains to be uncovered and future experiments, both wet lab and computationally, are needed to drive related discoveries. We believe that the novel tool DistAMo introduced here will help to uncover many more interesting patterns of DNA motif distributions which not only create scientific questions but also guides the search for answers. In addition, DistAMo might help to define chromosome construction rules for the growing field of synthetic genomics (Gibson et al., 2010; Annaluru et al., 2014; Messerschmidt et al., 2015; Schindler and Waldminghaus, 2015).

## Author contributions

TW and PS designed the study. PS, WH, and MS implemented the software and LJ and AG the web-based server version. TW and PS wrote the manuscript.

## Funding

This work was supported within the LOEWE program of the State of Hesse. We acknowledge technical assistance by the Bioinformatics Core Facility/Professorship of Systems Biology at JLU Giessen and access to resources financially supported by the BMBF grant FKZ 031A533 within the de.NBI network.

### Conflict of interest statement

The authors declare that the research was conducted in the absence of any commercial or financial relationships that could be construed as a potential conflict of interest.
