# Supplementary material for: DistAMo: A Web-Based Tool to Characterize DNA-Motif Distribution on Bacterial Chromosomes
Source: Front Microbiol. 2016 Mar 11;7:283. doi: 10.3389/fmicb.2016.00283 (PMC4786541; doi:10.3389/fmicb.2016.00283)
Supplement: Supplementary file 3 [file Image1.PDF]

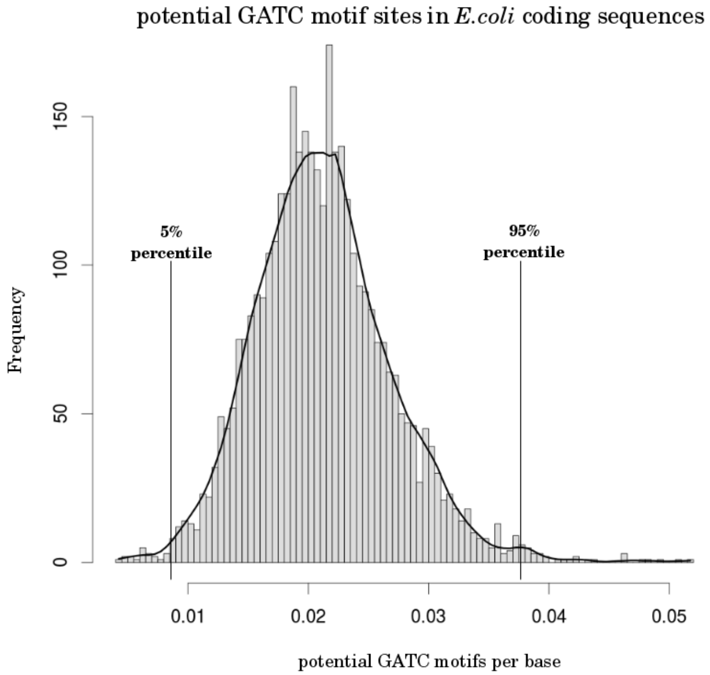
potential GATC motif sites in E.coli coding sequences
Frequency
0
50
100
150
5%
percentile
95%
percentile
0.01
0.02
0.03
0.04
0.05
potential GATC motifs per base

Distribution of potential motifs per base in *Escherichia coli*.
The number of sites potentially encoding a GATC motif without changing the amino acid sequence were counted for all proteins. The number of sites was normalized by the gene length to get the potential motif density (the number of potential motifs per base). The plot depicts the distribution of the potential motif density in *E.coli* proteins. From the 5% percentile to the 95% percentile of the distribution there is a four-fold difference in potential motif density, showing the high diversity the potential of proteins to be encoded by motif containing sequences.
