# Supplementary material for: DistAMo: A Web-Based Tool to Characterize DNA-Motif Distribution on Bacterial Chromosomes
Source: Front Microbiol. 2016 Mar 11;7:283. doi: 10.3389/fmicb.2016.00283 (PMC4786541; doi:10.3389/fmicb.2016.00283)
Supplement: Supplementary file 4 [file Image2.PDF]

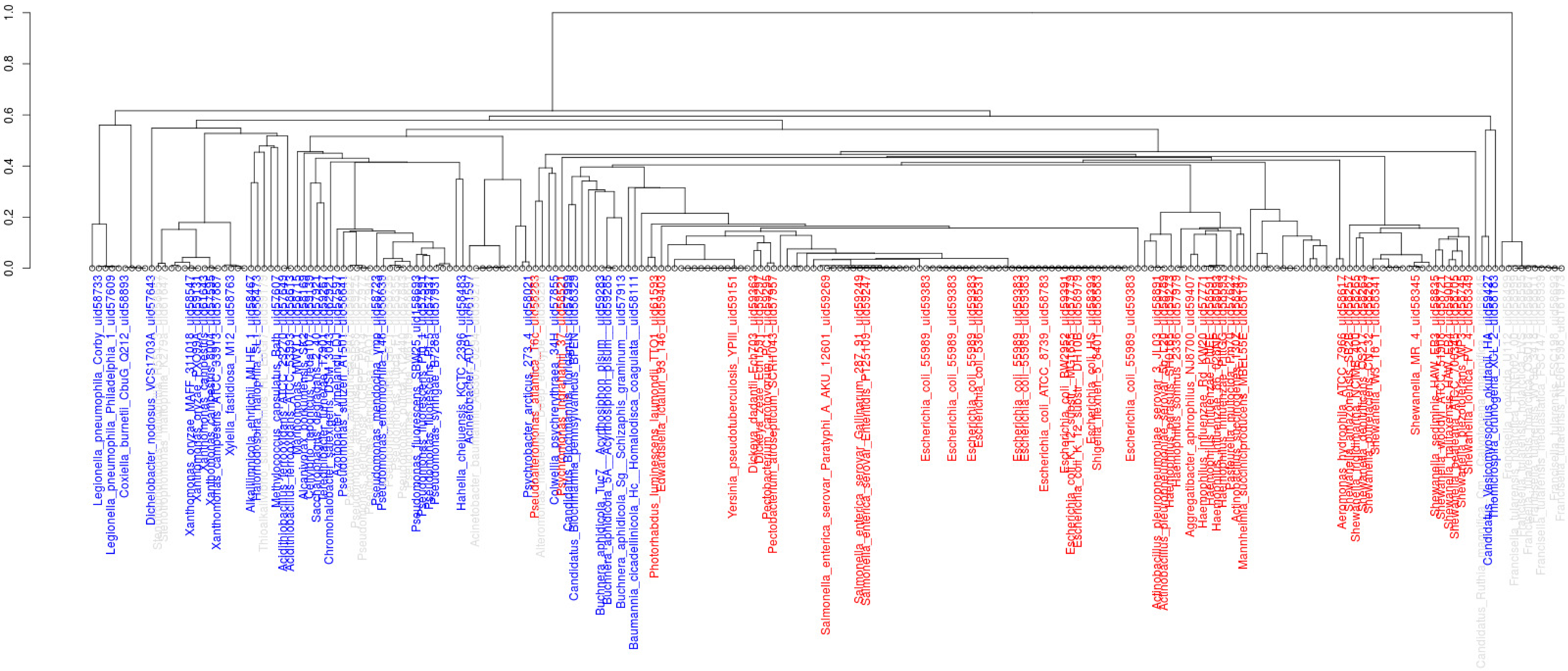

1.0
0.8
0.6
0.4
0.2
0.0
Legionella_pneumophila_Corby_uid58733
Legionella_pneumophila_Philadelphia_1_uid57609
Coxiella_burnetii_CbuG_Q212_uid58893
Dichelobacter_nodosus_VCS1703A_uid57643
Xylella_fastidiosa_M12_uid58763
Hahella_chejuensis_KCTC_2396_uid58483
Psychrobacter_arcticus_273_4_uid58021
Colwellia_psychrerythraea_34H_uid57855
Buchnera_aphidicola_Sg__Schizaphis_graminum__uid57913
Baumannia_cicadellinicola_Hc__Homalodisca_coagulata__uid58111
Yersinia_pseudotuberculosis_YPIII_uid59151
Salmonella_enterica_serovar_Paratyphi_A_AKU_12601_uid59269
Escherichia_coli_55989_uid59383
Escherichia_coli_ATCC_8739_uid58783
Aggregatibacter_aphrophilus_NJ8700_uid59407
Shewanella_MR_4_uid58345

Phylogenetic tree of γ-proteobacteria.
Dam positive (red) and Dam negative (blue) species included in this study are color-coded. The tree structure is taken from http://www.cbrg.ethz.ch/research/orthologous/speciestrees.
