# Supplementary material for: DistAMo: A Web-Based Tool to Characterize DNA-Motif Distribution on Bacterial Chromosomes
Source: Front Microbiol. 2016 Mar 11;7:283. doi: 10.3389/fmicb.2016.00283 (PMC4786541; doi:10.3389/fmicb.2016.00283)
Supplement: Supplementary file 5 [file Image3.PDF]

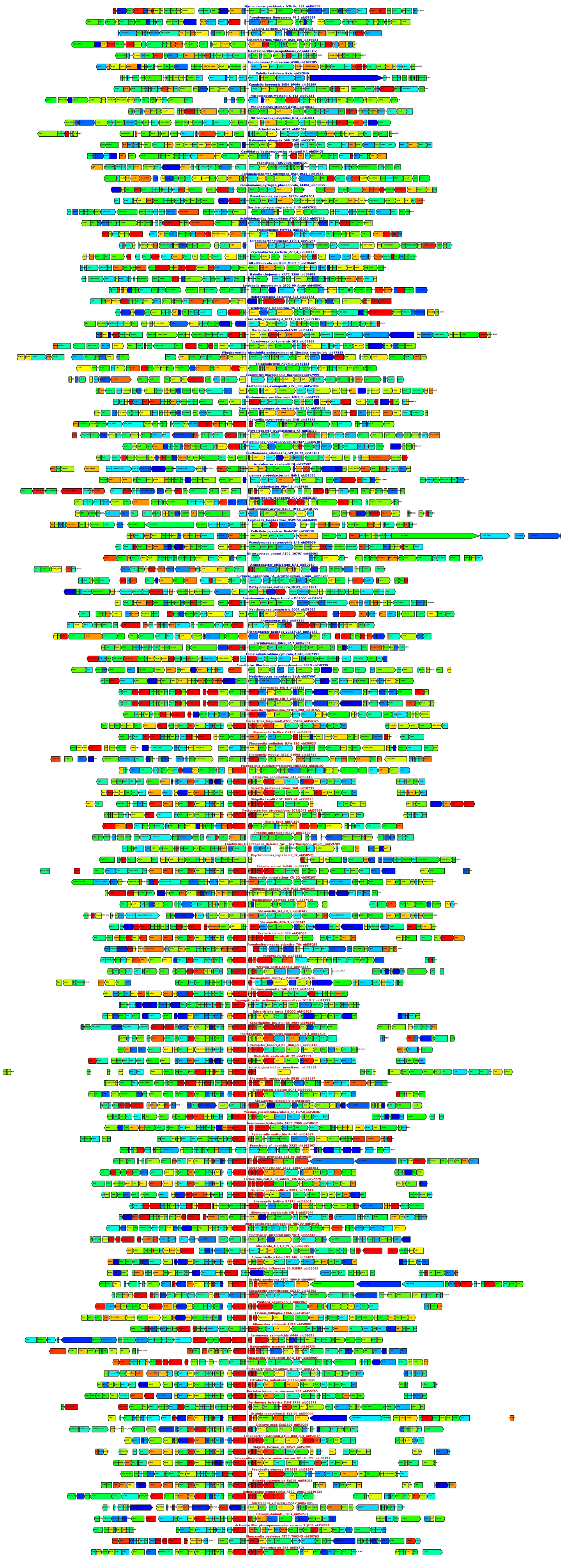

Gene arrangement of the oriC proximal region for all investigated genomes.
Gene names of *E.coli* orthologs are indicated. The GATC over- and underrepresentation is color coded in red and blue respecively. Species are grouped by Dam positive and negative indicated by a red and blue species label respectively. All regions were aligned and oriented according to the similarity to the *E.coli* core origin sequence (excluding flanking coding sequences).The position of the origin of replication is indicated by a vertical grey line in the center.
